# Biology of Porcine Parvovirus (*Ungulate parvovirus 1*)

**DOI:** 10.3390/v9120393

**Published:** 2017-12-20

**Authors:** István Mészáros, Ferenc Olasz, Attila Cságola, Peter Tijssen, Zoltán Zádori

**Affiliations:** 1 Institute for Veterinary Medical Research, Centre for Agricultural Research, Hungarian Academy of Sciences, 1143 Budapest, Hungary; olasz.ferenc@agrar.mta.hu (F.O.); zadori.zoltan@agrar.mta.hu (Z.Z.); 2 Ceva-Phylaxia Zrt., 1107 Budapest, Hungary; attila.csagola@ceva.com; 3 INRS-Institut Armand-Frappier, Université du Québec, Québec, QC H7V 1B7, Canada; Peter.Tijssen@iaf.inrs.ca

**Keywords:** *ungulate protoparvovirus 1*, porcine circovirus type 2, viral entry, nuclear localization signal, VP2 trimer, genetic diversity, mutation rate

## Abstract

Porcine parvovirus (PPV) is among the most important infectious agents causing infertility in pigs. Until recently, it was thought that the virus had low genetic variance, and that prevention of its harmful effect on pig fertility could be well-controlled by vaccination. However, at the beginning of the third millennium, field observations raised concerns about the effectiveness of the available vaccines against newly emerging strains. Subsequent investigations radically changed our view on the evolution and immunology of PPV, revealing that the virus is much more diverse than it was earlier anticipated, and that some of the “new” highly virulent isolates cannot be neutralized effectively by antisera raised against “old” PPV vaccine strains. These findings revitalized PPV research that led to significant advancements in the understanding of early and late viral processes during PPV infection. Our review summarizes the recent results of PPV research and aims to give a comprehensive update on the present understanding of PPV biology.

## 1. Introduction

PPV (Porcine Parvovirus (*Ungulate parvovirus 1* in the *Protoparvirus* genus) was first recognized as a member of the *Parvoviridae* family and causative agent of SMEDI syndrome (stillbirths, mummification, embryonic death, and infertility) at the end of the 1960s [1,2,3]. Although SMEDI had been described a few years earlier [4], the causative agent of the disease was first erroneously identified as a picornavirus. Since its discovery, PPV has remained a constant worldwide problem of the pig industry, still being one of the most common and important infectious agents of infertility. In this review, we concentrate on the results of the last 15 years and try to give an overview of the research that has supplied relevant information about the biology and containment of the virus.

## 2. Pathogenesis

In most cases, PPV infection alone does not cause clinical symptoms in non-pregnant adult pigs or piglets. Strain virulence is defined by the severity of the reproductive failure it can cause [5].

**Figure 1:**
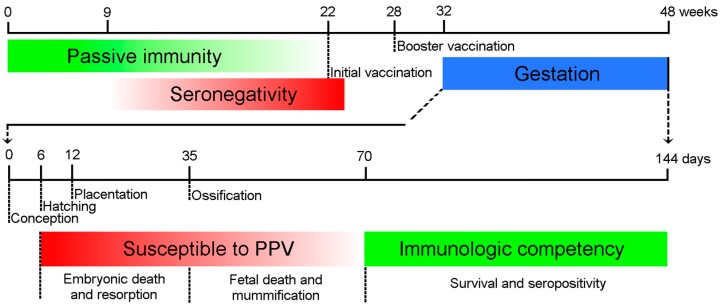
Major time points of Porcine Parvovirus (PPV) infection and immune response. The maternal antibodies protect the piglets passively (green line) until 9–22 weeks of age. The animals should be inoculated first after the depletion of maternal antibodies, and long-term immunity is maintained by booster vaccination. The embryos are susceptible to the infection (red line) until the Development of immune competence around day 70 day of gestation (green line). The consequences of the intrauterine infection depend on the time of the infection.

The outcome of the infection in the fetus varies with the progression of gestation in sows. Experimental and epidemiological studies indicate that PPV infection during the first half of pregnancy can lead to reproductive failure [6,7,8,9,10,11]. Immunocompetent fetuses infected after day 70 of gestation develop an antibody response and usually survive the infection. Vertical transmission to the fetus takes 12 to 18 days after the initial infection of dams by the natural (oral) route [7,8] and somewhat less time by intramuscular injection. Consequently, infection of sows after 56 days of gestation usually does not cause damage to the fetus [12] (Figure 1).

Besides the timing, the genetic makeup of the virus has a determining effect on the result of the fetal infection. Low pathogenic and vaccine strains (e.g., NADL-2 and MSV) cannot cross the placental barrier as efficiently as highly pathogenic strains (e.g., Kresse and 27a) [5,13], so their harmful effect on gestation cannot be detected as frequently as that of the highly pathogenic strains. However, direct injection of NADL-2 into the amnionic fluid can lead to fetal death [14,15].

One of the unanswered questions about PPV-induced reproductive failure is how the virus passes the placental barrier. No evidence was found about the replication of the virus in the uterine epithelium or in the trophectoderm, so the “replicate through the barrier” theory seems improbable. Alternatively, it was suggested that PPV invades the fetus in or on the surface of maternal macrophages. This is hypothesized given that a high number of macrophages reside within the endometrium/placenta during the entire period of gestation, and monocytes and peritoneal macrophages were shown to phagocytize NADL-2 (though they do not support its replication) [16]. In fact, any direct evidence for macrophages crossing from mother to fetus is also missing.

Experimental infections of pregnant sows indicate that more than 10,000 times as much of strain NADL-2 is needed to reach the conceptus transplacentally as of virulent NADL-8 to establish infection [14]. Applications of nucleic acid detection methods revealed significant differences in the distribution and the quantity of viral DNA in embryos infected with different strains. Kresse and NADL-8 were detected by in situ hybridization from the liver, while only Kresse was detected from brain and spleen [17]. Highly virulent 27a was found in high titer (>10^11^ copy/10^6^ cell) in all 10 organs examined by real-time PCR, while less virulent field (143a) and vaccine strains (NADL-2 and MSV) showed limited tissue distribution and were mainly detected in the kidneys, with much lower titers (~10^3^ copy/10^6^ cell) [15]. These observations suggest that strain differences in tissue specificity, and consequently, in infection-initiating capability in the embryo, might also play a role in the outcome of fetal infection.

## 3. Virus-Cell Interaction

The in vivo target cells of PPV in neonates and older animals are difficult to determine. The virus can replicate in activated lymphocytes, cannot replicate in blood monocytes, and the results concerning replication ability in macrophages are inconsistent [18,19]. Based on PCR experiments, PPV can propagate in the cells of heart, lung, kidney, spleen, endometrium, and small intestine [20,21]. However, PCR cannot distinguish between the viruses that are produced in the organs, and viruses that are transported there by the vascular system. This may explain the controversial results of detecting PPV in the lymphoid nodes [20,21].

As the first step of entry, the virion binds to terminal sialic acid moieties of the glycoproteins on the cell surface. Both *O*- and *N*-linked sialic acids are used for attachment as proved by the rebuilding of neuraminidase-digested sialic acid moieties (digested by (2,3)-*O*-sialyltransferase and (2,3)-*N*-sialyltransferase) [22]. Studying entry by inhibitors revealed that besides clathrin-mediated endocytosis and macropinocytosis, a third unknown entry mechanism is likely to be involved in PPV penetration, while the caveolae route does not play any role [22]. PPV has a tendency to aggregate below pH 8. Single particles strongly prefer entry by clathrin-mediated endocytosis, whereas aggregates clearly favor macropinocytosis [22].

The endosomal translocation to the late endosomes or lysosomes, and acidification between 2 and 10 h p.i. seems to be crucial for effective PPV infection [22]. Acidification likely contributes to the externalization of the unique part of the capsidprotein 1 (VP1up) that is normally internalized in mature protoparvovirus particles [23,24]. The VP1up contains a phospholipase A2 (PLA2) domain that plays a crucial role in viral release from the endosomes to the cytoplasm, acting via enzymatic digestion of the membrane phospholipids that destabilize the endosomal membranes [24,25]. This biomimicry of host cellular processes is not unique to parvoviruses. For instance, caliciviruses use cholic acid to activate acid sphingomyelinase (ASM), which cleaves sphingomyelin on the inner leaflet of endosome membranes and produce ceramide. Increased ceramide destabilizes membrane integrity by forming channels or causing lipid flip-flop. This allows the virus to escape [26]. As co-precipitation and inhibitor studies show, ubiquitination and interaction with proteasomes in the cytoplasm are indispensable steps for effective PPV infection, despite the fact that protease degradation of the capsids could not be demonstrated during entry. In the movement of viral particles towards the nucleus, both the microtubule and the actin networks are involved. Microtubules are crucial in the first 8 to 10 h of the infection, suggesting that they have a role in the endosomal transport of PPV to the perinuclear region. Actin activity is needed later (up to 12 to 16 h p.i.) for productive infection, and it is most probably necessary not only for the transport of incoming viruses, but also for the nuclear transport of the newly synthesized proteins [22].

**Figure 2:**
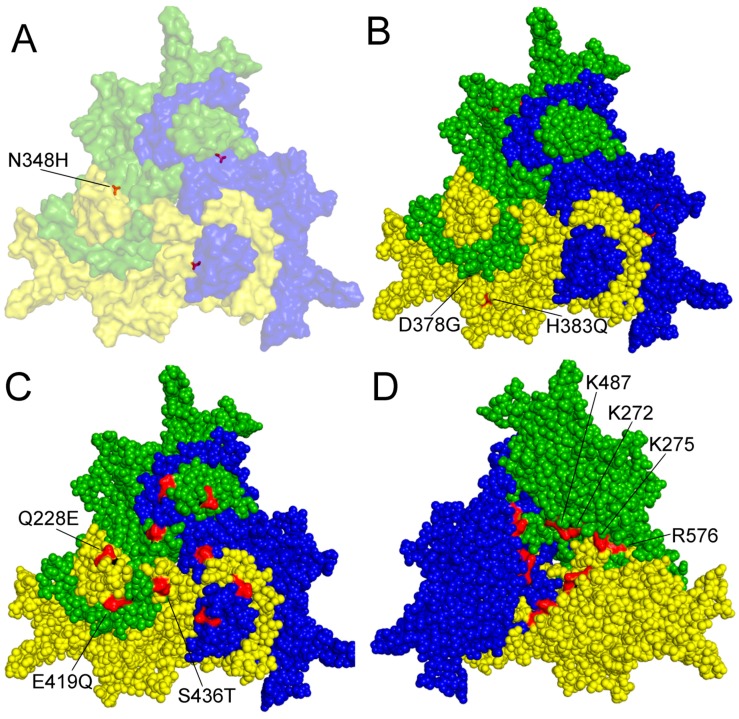
Amino acid residues with known function on the surface of a capsid protein 2 (VP2) trimer of PPV. A–C outer surface is shown. (**A**) amino acid (aa) 348 highlighted contributing to P2 replication in the canine cell line A72; (**B**) aa 378 and 383 are involved in tissue tropism and probably in virulence; (**C**) aa 228E, 419Q and 436T are characteristic of the members of the D cluster; (**D**) aa K272, K275, K487 and R576 form a nuclear localization motif on the inner surface of the trimer. Numbering is presented according to NADL-2 VP2 sequence, amino acid changes labeled by commonly used colloquial nomenclature. Trimer was generated by Viper program and visualized by Polivew.

From the five linear basic clusters identified in the VP1up, only three were confirmed to serve as nuclear localization signals (NLS) [27]. The first, located at the amino terminal of the VP1up (from third to ninth amino acid (aa)) is a classic Pat7 NLS, while the other two (between the 122nd and 137th aa) comprise a classic bipartite NLS. Both NLSs are essential for viral replication. Their mutations do not affect viral assembly but abolish productive infection, strongly suggesting that they are responsible for the nuclear transport of the incoming virion in the early phase of infection [27]. Just like in the case of minute virus of mice (MVM), the VP2 of PPV assembles into trimers in the cytoplasm [27,28]. A non-linear nuclear localization motif (NLM) is comprised of four amino acids (K272, K275, K487, and R576) (Figure 2D) on the inner surface of the trimers, and is recognized and transported to the nucleus by the import machinery of the cells, where they assemble to a capsid. It seems that in all protoparvoviruses, both VP1 NLS and VP2 NLM are internalized in the nucleus during viral assembly, and become inaccessible for transporter proteins [27]. Interestingly, MVM was shown to be actively transported out of the nucleus [29]. It is therefore tempting to speculate that the presence of NLM or NLS on the particle would interfere with such transport, and that would be the reason for the concealment of these signals. However, in the case of PPV (and many other protoparvoviruses), there was no sign of any vesicular transport of the assembled virions towards the cell membrane, leaving the question about the functional role of this topological phenomenon open.

Most of the known mutations influencing the biological feature of PPV were found on the capsid protein. The only documented exception is the I481L mutation in the nonstructural 1 (NS1) protein of the NADL-2 strain, which, together with the N348H change of VP2, contributed to the ability of the P2 strain to cause a cytopathic effect and replicate with high titer in the canine cell line A72 [30] (Figure 2A).

The genomes of NADL-2 and Kresse differ by 13 nucleotides (nt) and a 127-nt repeated sequence near the right-end hairpin. There are only six aa differences between the structural proteins of the two viruses, of which five are also present in other virulent field strains (I215T, D378G, H383Q, S436P and R565K). Three of these (D378G, H383Q and S436P) localizing on the capsid surface was enough to abolish NADL-2 replication in primary bovine testis cells (TV) and reduce titer and cytopathic effect to the level of Kresse in cell lines with porcine origin (PT and PFT) [31]. Later, S436P was found not to be involved in tissue tropism in vitro [32]. Since threonine can be detected in position 436 in virulent (27a) and avirulent (143a) strains, this raises the possibility that mutations of aa 378 and 383 of VP2 (Figure 2B) might be enough to modify the pathogenicity of PPV in vivo [5].

More detailed studies with chimeras verified that mainly the capsid proteins determine viral replication efficiency in both porcine and bovine cell lines. However, an interaction between the NS1 protein of the VP coding region and the noncoding repeated sequence may occur and may influence viral replication, most probably through the low-affinity NS1 binding sites scattered throughout the genome. The NADL-2 capsid sequence and repeat region are favorable compared to that of Kresse regarding viral fitness and replication efficiency in vitro [32,33]. However, this is obviously not the case in vivo.

PPV infection facilitates the accumulation of total cellular p53 as early as 3 h p.i. in PK-15 cells (origin form porcine kidney). In the infected cells, p53 activates caspase-9 and caspase-3 through the mitochondria-mediated apoptotic pathway, releasing cytochrome c from the mitochondria [34]. The ratio of apoptotic cells can reach 50% [34,35] in YL strain-infected swein testis (ST) and PK-15 cells at the late phase of infection (60 h p.i.), while in Kresse-infected PT cells, the number of apoptotic cells remains below 14% (as indicated by the number of pyknotic and karyorrhectic cells). Swelling of the infected nuclei, early cell membrane failure (as shown by propidium-iodide uptake), rapid lactate dehydrogenase and free viral DNA release, all point toward necrosis as the main form of cell death in PT cells during Kresse infection [36]. Even subtle mutations of the PPV capsid can modify interactions with host factors and can change the cytopathic effect of the virus [31,33]. Although it is difficult to compare the results of the different experiments that investigated the cytopathic effect of PPV infection, the emerging picture is that it can activate the necrotic and/or apoptotic pathways just like other protoparvoviruses [37], and the actual outcome of the infection largely depends on the viral strain and the cell type. Nevertheless, early cell disintegration accelerates the release and the spread of PPV in vitro. These processes must have highly significant effects on the virus life cycle in vivo, because a small alternatively translated protein (SATp) has evolved in PPV (and other protoparvoviruses) to facilitate quick cell lysis and virus release. The protein is expressed by a leaky scanning mechanism from the same mRNA as VP2, and its start codon is seven nucleotides downstream of the VP2 initiation codon. SATp is an endoplasmic reticulum (ER) resident short membrane protein (68aa) that contains a single membrane-spanning helix and accelerates cell death and viral spread [38]. PPV infection induces an unfolded protein response (UPR) in infected PT cells, regardless of the presence or absence of SATp. It also leads to the activation of the anti-apoptotic, reversible ER stress marker Xbp1 (from 12–14 h p.i.). The marker Xbp1 most probably delays cell death to allow viral synthetic processes to be completed. However, in a later phase, the presence of SATp accelerates cell death by making ER stress irreversible, as shown by the higher rate of expression and the nuclear localization of CHOP (from 22 h p.i.). The involvement of severe ER stress in porcine testis (PT) cell necrosis and viral egress was confirmed by the treatment of infected cells by ER stress-inducing chemicals (MG132, dithiothreitol, and thapsigargin), which accelerated the egress and spreading of both the wild-type and the SAT^−^ viruses [36].

## 4. Genetic Variation and Evolution

Until the beginning of the 2000s, genetic changes of the PPV genome were not studied systematically due to field observations that the virus remains very stable immunologically, and commercial vaccines developed from ancient strains provide full protection against newly emerging PPV variants. Studies in the first decade of the 21st century seemed to reinforce the conventional view, and even suggested that PPV has a more conservative genome than other parvoviruses [39,40,41].

However, systematic studies in the last fifteen years concentrating on the genetic diversity in VP proteins in field isolates from domestic pigs revealed at least seven clusters, with a predominance of the European strains in clusters C and D, and Chinese strains in cluster F [42].

A similar correlation between clustering and geographical distribution of strains isolated from wild boars was not observed. Fourteen viruses from Romanian wild boars could be distributed into five clusters that grouped together with domestic isolates from all over the world [43]. This finding indicates that PPVs of wild boar populations are more diverse than viruses of domestic pigs in the same area. It also suggests that the pressure driving PPV evolution in domestic pig and wild boar population are fundamentally not so different, wild boar- or domestic pig-specific PPV strain clusters have not yet evolved, and/or viruses more or less freely shuttle between subspecies [43]. Field observations and experimental investigations of some of the new highly virulent isolates of cluster D revealed that antisera raised against “old” PPV vaccine strains cannot effectively neutralize these viruses [5,44]. It was hypothesized that these mutants might have emerged by escaping immune pressure forced by vaccination. However, neither in vitro nor in silico studies could find any evidence to support this idea. A decrease in genetic diversity of PPV was observed in the presence of antibodies in tissue culture or in vaccinated herds as modeled from available sequence data. Mutations found on immune selected capsids (NADL-2 I320S, H383Q; Str. Challenge S45T, P436S) were not present on novel variants either [45]. The authors concluded that vaccine failures and non-vaccinated animals (e.g., wild boars) may have a more important impact on the emergence of new phenotypes than vaccinated populations.

No obvious correlation could be observed between clustering (phylogeny) and virulence status of PPV isolates. Closely related highly virulent and less virulent strains can be found in most of the clusters [5,14,42], suggesting that the ability of field strains to effectively cross the placental barrier and kill the fetus has a secondary role on viral spreading and PPV evolution. It seems that other, less studied biological features (e.g., tissue specificity and long-term/high titer shedding), which in certain cases may be directly or indirectly influenced by virulence, determine the fitness of a PPV strain under highly variable field conditions.

The application of molecular clock models in independent investigations predicted that current strains are the result of relatively recent evolutionary events, and main branches started to diverge from each other in the last 10–60 years [42,43,46]. However, it remained elusive how the predicted sole ancestor of the present strains evolved prior to 60 years ago. Another analysis estimated the age of the most recent common ancestor of PPV stains to be around 250 years, and suggested that Western colonialization could have contributed to the spread of PPV [47].

Investigations of the mutation patterns of the *NS* and *VP* genes of field strains revealed the contrasting evolution of the two coding regions. The mutation rate of VP is 30–50 times higher (3–5 × 10^−4^ mutation/nucleotide/year) than that of one of the *NS* genes (10^−5^) [46,47]. This, together with the negative difference between non-synonymous and synonymous substitution rates (dN–dS) found in the NS region, suggests that the number of nucleotide changes that allow the sustained functionality of the NS proteins is limited, and that purifying selection determines the evolution of the region. In contrast, earlier investigations suggested that the VP1/VP2 gene evolves under a near-neutral model (drift) [48], but several positions are under positive selection, especially on the outer loops of the capsid that determine cellular interactions and immunogenicity [46,47,48]. In addition, the majority of mutations were found to be in the surface loops [46,48,49,50], among them, 215, 228, 383, 414, 419, and 436 seem to be the main variable sites in the capsid [47]. However, more recent investigations on higher numbers of samples have given a more nuanced picture, finding no mutation hotspots between loops and β-strands [42], and showing that complete VP1 is also under purifying selection [42,43,47].

A three aa surface substitution is characteristic of the members of cluster D (Q228E, E419Q, and S436T), including 27a (Figure 2C). Position 228 is part one of the nine known linear epitopes on VP2 [51]. However, the contribution of these aa’s to the apparent immune escape feature of 27a remains to be studied [5].

Recombination does not play a major role in the evolution of PPV [42,47].

It is a widely accepted theory that the main reason behind CpG depletion in small DNA viruses is due to natural selection coming from replicative advantage and/or immune escape [52,53]. The PPV genome is CpG-depleted, yet neither experimental nor in silico investigations can confirm these assumptions in the case of PPV. The lack of measurable biological effect after introducing additional CpGs into the PPV genome argued against the replicative advantage of CpG depletion, and the ascendant distribution of CpGs by position does not support the presence of immunological pressure against CpGs. These data, taken together with the findings that CpG sites are more prone to be mutated than GC or C and G sites in the PPV genome, suggest that mutational pressure, rather than a selective force, is responsible for CpG underrepresentation in the PPV genome [54].

## 5. Immunity and Prevention

Hardly anything is known about the role of cellular immunity in controlling PPV infection. The only investigation made in this direction found weak cytotoxic T cell (CTL) activity, suggesting that T-cell activation may occur with repeated exposure to the virus, but confirmed that effective clearance of PPV infection is obtained by rapid antibody response in infected pigs [55].

Many immunological studies proved that the presence of neutralizing serum antibodies is a decisive factor in the outcome of the PPV infection [5,44,55,56]. Since the placenta is impermeable to maternal antibodies, neonatal piglets obtain passive protection against PPV1 by taking up maternally derived antibodies (MDA) from the colostrum. A strong correlation can be observed between MDA levels in piglets and the anti-PPV colostrum antibody levels in their dams. The amount of MDA decreases steadily in piglets as their age progresses. Previous studies have shown that MDA levels last up to 14–26 (mean 21) weeks in pigs [7]. Etoh et al. [57] found that a high titer of MDA after birth may persist until the 22nd week, while the low level of MDA can be exhausted within nine weeks. A recent study has demonstrated that maternal protection terminates somewhat sooner [58]. This investigation has found that PPV1-specific antibodies are detectable in 93.6% of the one-week-old piglets after colostrum uptake. At 57 days of age, 35.3% of pigs carry detectable amounts of PPV1-specific antibody, while at 87 days only 1.5% of pigs do the same. These values have been verified by a field survey conducted in a Hungarian herd (Cságola personal communication).

High levels of passive antibodies can prevent infection, while lower levels can reduce dissemination of PPV from infected pigs [56,59]. Nonetheless, in a PPV-affected, unvaccinated herd, sooner or later the majority of the piglets will be infected with the virus by lateral transmission [58] as indicated by the high occurrence of seropositive gilts (86%) [58]. Despite the presence of antibodies at high titer, relatively high virus copy number (>10^4^/mL) can be detected in some animals until day 21 p.i., and viremia can endure until day 42 p.i. [60]. Another systematic experiment indirectly supports this observation, finding that infected pigs can shed the virus in body excretions (e.g., feces, nasal discharge) from the 4th day p.i. for at least 49 days, though the quantity significantly decreases after two weeks [61].

PPV infection activates IFN-γ and IFN-α synthesis [60,62], and the seronegative pigs start antibody production shortly after infection. PPV-specific antibodies can be detected as early as day 6 p.i. [60], and the antibody titer peaks on days 14–21 [3,12,63,64]. An in vitro study demonstrated that the transcription of the potent B cell differentiation factor IL-6 is stimulated through the toll-like receptor 9 (TLR9)-mediated NF-κB signaling pathway in infected cells. It is additionally suggested that IFN-α might also be activated through these pathways during infection. The expression levels of 17 immune-related miRNAs, including miR-10b, miR-20a, miR-19b, miR-181a, miR-146b, and miR-18a were found to be significantly altered in PK-15 cells during PPV infection. These miRNAs were shown earlier to be involved in the regulation of at least six immune response pathways, TLRs and NF-κB among them [65].

The inactivated vaccines used currently are based on NADL-2 (cluster A) and similar strains, and were isolated 40 years ago. These vaccines are effective against homologous infections, but do not prevent infection and virus shedding after challenge with the antigenically heterologous 27a (cluster D) strain [44]. However, they can protect the fetus against disease. Infection of 27a in pigs or the inoculation of the virus into rabbits induced 100- to 1000-fold lower homologous neutralizing antibody titers than heterologous titers against 143a, NADL-2 or MSV strains [5]. Vaccination by inactivated 27a prevented fetal death after homologous virus challenge with PPV-27a. However, a substantial increase in antibody titers was detected after infection, indicating virus replication in the immunized animals [61]. These experiments suggest that 27a has unique immunological features, and although present vaccines are able to prevent disease, inactivated vaccines cannot induce the desired sterile immunity against 27a [61]. Modified live-virus or vector vaccines inducing cellular immunity might be alternative approaches. However, due to the past success of the inactivated vaccines and the complication and cost of licensing genetically modified organisms, only a few attempts were made in this direction. Genetically modified Lactobacillus and pseudorabies virus expressing the PPV VP2 protein were shown to induce neutralizing antibodies against PPV, but in vivo challenge experiments for testing vaccine efficacy are still missing [66,67].

## 6. Co-Infection with Circovirus

The post-weaning multisystemic wasting syndrome (PMWS) was first identified in western Canada in the 1990s [68]. Although not all Porcine circovirus type 2 (PCV2) infected animals develop disease symptoms, it was demonstrated that PCV-2 alone could induce the clinical signs of PMWS in cesarean-derived, colostrum-deprived or specific pathogen free (SPF) pigs [69,70]. Laboratory experiments and field studies have shown that co-infection with PPV and other pathogens potentiates the effect of PCV-2 in the development of PMWS [71,72,73,74]. However, even simultaneous co-infection of SPF piglets with PPV and PCV-2 [62,75,76] does not necessarily lead to the manifestations of PMWS. The age, management factors, immunological status, and the time of infection can significantly modify the outcome of the co-infection [62]. Early PCV-2 (prenatal or colostral transmission) infection followed by PPV over-infection within a few weeks seems to significantly increase the onset of PMWS in serologically negative animals [75,77]. However, serological analysis of both experimentally-infected and field samples suggests that the presence of serum antibodies against any of the two viruses reduce the risk of developing PMWS [76,78].

PPV might facilitate PCV-2 infection either indirectly, by reducing immunoprotection or/and stimulating virus replication by host cell activation, or directly, by promoting virus DNA replication infecting the same host cells. In fact, both PCV-2 and PPV were shown to replicate in lymphocytes [18,19,79] and detected in (inguinal) lymph nodes during co-infection [75]. However, the indirect involvement of PPV seems more probable given that non-viral immunostimulation of PCV-2-infected animals also promotes PMWS, over-infection with PPV seems more effective to induce the disease than co-infection [62,75,76], and PPV has been associated with transient immunosuppressive effects [17,18,19].

## 7. Detection and Isolation of PPV

The virus can agglutinate chicken, guinea pig, mouse, human, monkey, rat, and cat erythrocytes. Thus, hemagglutination inhibition assays (HAI) were developed relatively early [64,80] and they are still used in research and practice in some countries [5,44,81,82]. Other serological methods like the serum neutralization (SN) assay or the modified direct complement-fixation (MDCF) test were also utilized earlier, but today the enzyme-linked immunosorbent assay (ELISA) is the most frequently applied test to detect PPV-specific antibodies. A recombinant NS1 protein-based DIVA test (Differentiating Infected from Vaccinated Animals) was also developed to distinguish vaccinated (inactivated vaccine) from infected pigs [83].

Sensitive nucleic acid detection methods can provide accurate information about the presence and the quantity of the virus in the animals. Swab (nasal, rectal, genital tract), blood, kidney, lung, lymph nodes and tissues from abortion material are used most frequently for DNA preparation. Using real-time PCR, a detection limit of 20–500 copies could be achieved [15,84,85,86]. The loop-mediated isothermal amplification (LAMP) assay, nanoPCR, and recombinase polymerase amplification (RPA) assay, were also successfully applied for PPV detection with a very low detection limit (5, 56, 300 copies per reaction respectively) [87,88,89]. However, the application of these methods in the everyday practice is rare or nonexistent. Recent diagnostic developments focus on the detection of several porcine pathogens in one PCR tube, including RNA viruses. PPV can be detected together with the pseudorabies virus, classical swine fever virus, African swine fever virus, porcine reproductive and respiratory syndrome virus, porcine circovirus type 2, or Japanese encephalitis virus in multiplex (reverse transcriptase) PCRs [90,91,92,93,94,95,96].

For the isolation of PPV established cell lines, such as swine testis (ST and PT), pig fallopian tube (PFT) and pig kidney (PK-13, PK-15) cells can be used [2,97]. Though some PPV isolates are able to replicate in Cos7 (African green monkey) [27], KB (human) [98], or A72 (canine) cells [30], porcine cells are much more susceptible than cell cultures originating from other species. Depending on the strain, 500–10,000 packaged genome copies are needed to initiate infection in PT cells [36,38], which seem to be the most sensitive to PPV infection [31]. Contaminated trypsin, derived from the pancreas of PPV-infected pigs, was a major cause of virus transmission into cell cultures before suppliers started to test for the presence of PPV. This was probably also the case for the KBSH strain isolated from KB cells. This strain was one of several parvoviruses recovered from permanent human cell lines [98].
